# Empyema necessitans due to aspergillus in a 3-year-old paediatric patient at a referral hospital in Nairobi, Kenya

**DOI:** 10.11604/pamj.2019.34.86.19469

**Published:** 2019-10-13

**Authors:** Anne-Marie Macharia, Loice Achieng Ombajo, Abdirahman Hashi Farah, Edwin Oloo Walong, Gladys Nthambi Mwango, Dalton Wamalwa

**Affiliations:** 1 Unit of Clinical Infectious Disease, Department of Clinical Medicine and Therapeutics, University of Nairobi, Nairobi, Kenya; 2 Department of Paediatrics and Child Health, University of Nairobi, Nairobi, Kenya; 3 Department of Human Pathology, University of Nairobi, Nairobi, Kenya; 4 Department of Diagnostic Imaging and Radiation Medicine, University of Nairobi, Nairobi, Kenya

**Keywords:** Invasive aspergillosis, child, empyema

## Abstract

We present a case of empyema necessitans due to aspergillus in a young child. The incidence of deep fungal mycoses in children, especially in developing countries is not known. There is paucity of data and access to diagnostics is usually limited. Our patient had received treatment for pulmonary tuberculosis which is endemic in Kenya for four months before diagnosis was made. We present this case to highlight the importance of considering alternative diagnosis when there is non-response to anti-tuberculous therapy.

## Introduction

We present a case of empyema necessitans due to aspergillus in a young child. The incidence of deep fungal mycoses in children, especially in developing countries is not known. There is paucity of data and access to diagnostics is usually limited.

## Patient and observation

**Figure 1:**
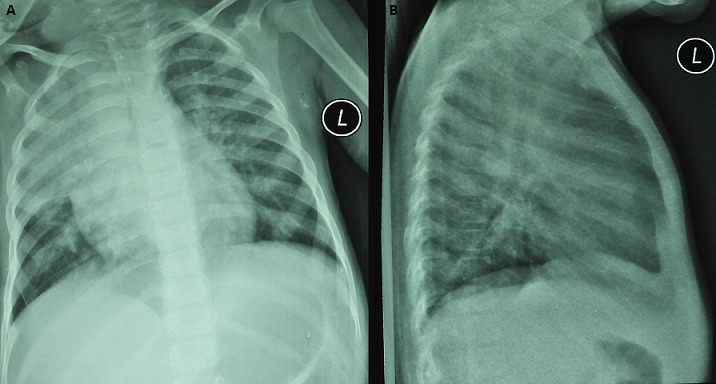
(A, B) AP and lateral chest radiographs prior to TB diagnosis show right upper lobe consolidation with volume loss, diffuse patchy and nodular infiltrates, right para-tracheal and left hilar adenopathy

A male child aged three years was referred to our hospital from a rural health facility. He had been initiated on empiric anti-tuberculous therapy with no clinical improvement after three months of treatment. The patient had been in good health until six months prior when he developed cough and intermittent fever. He was admitted with a clinical diagnosis of pneumonia for which unspecified antibiotic therapy was given with transient improvement. Similar symptoms recurred two months later and he was readmitted. A chest radiograph showed right upper lobe consolidation with volume loss, right para-tracheal and left hilar adenopathy and bilateral fine reticulonodular opacification (Figure 1 A, B). A Gene ^®^Xpert™(MTB/RIF) performed on fasting gastric aspirates was negative. A clinical diagnosis of pulmonary tuberculosis was made based on the algorithm for diagnosis of paediatric tuberculosis [1] and standard anti-tuberculous therapy (2HRZE/4HR) was initiated. His condition continued to deteriorate; he developed a mass on the right upper chest wall and was referred to our facility three months into tuberculosis (TB) treatment, for evaluation of suspected malignancy. At presentation, he had persistent fever and cough with dyspnea that disrupted feeding. The cough was scantily productive of white sputum. Progressive swelling had been noted on the right chest wall and there was marked weight loss. There was no recent travel history. There had been no contact with domestic livestock or birds nor with any person with tuberculosis. He was HIV negative. At presentation, temperature was 39.4°C, respiratory rate 40/min, pulse rate 125/min, blood pressure 100/70 mmhg, spO2 88%.

**Figure 2:**
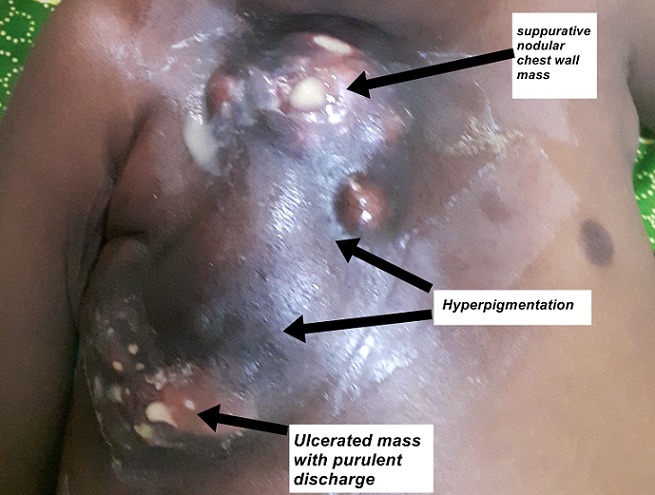
The right chest mass is multinodular with hyperpigmentation of the skin of skin, multiple ulcers and discharging sinuses

**Figure 3:**
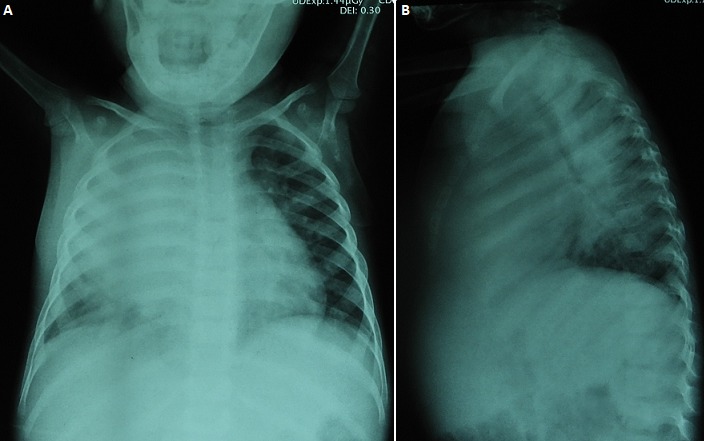
(A, B) AP Chest radiograph done after 3 months of anti-tuberculous therapy

**Figure 4:**
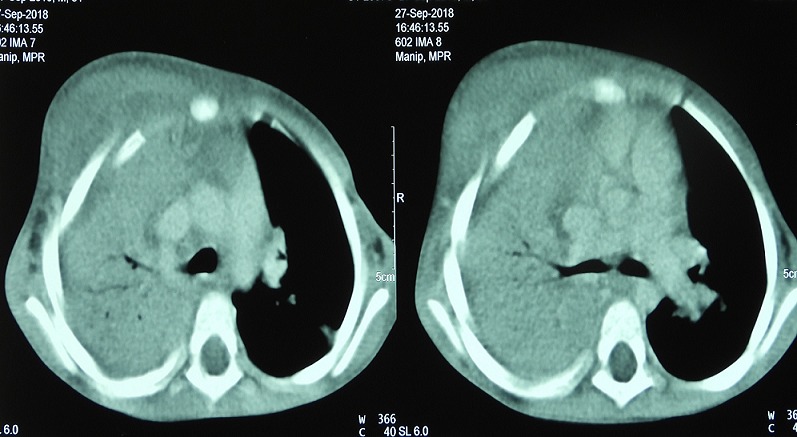
Contrast enhanced CT scans of the chest showing consolidative atelectasis of the right upper lobe with massive paratracheal adenopathy and chest wall mass. Enlarged axillary lymph nodes and erosion of the ribs are also noted

He had no conjunctival pallor or jaundice. He had multiple enlarged discrete right axillary lymph nodes, largest 2cm in diameter; firm, non-tender and mobile. He had a firm, tender, diffuse swelling on the right upper chest wall with fluctuant areas, that over subsequent days developed draining sinuses with no discharging granules (Figure 2). He was tachypnoeic with dullness to percussion and bronchial breathing in the right upper zones posteriorly. The rest of the systemic examination was normal. Serial results of laboratory test results are shown in Table 1. Blood cultures and pus swab cultures of the draining sinuses showed no growth. β D Glucan and galactomannan assays were not available. Chest radiographs taken on admission showed no improvement when compared to previous films (Figure 3 A, B). Contrast enhanced CT Scan of the chest was done with images as shown (Figure 4). An ultrasound guided fine needle core biopsy of the chest wall mass was carried out. Histology revealed necrotizing granulomatous inflammation with pieces of acutely branching hyphae morphologically consistent with *Aspergillus* sp. These were also demonstrated on Periodic Acid Schiff stained sections. Genomic testing and fungal culture were not performed. On admission the patient had been empirically started on ceftriaxone and vancomycin therapy; when the histology was received, treatment with amphotericin B was initiated and the patient was scheduled for surgical debridement and lobectomy. He developed respiratory failure and succumbed on the second day of treatment. The next of kin did not consent for an autopsy.

**Table 1:** Laboratory data

Variable (Units)	Reference Range Age adjusted	On admission	Week 2	Week 3
Haemoglobin (g/dl)	10.5 - 13.5	13.5	11.0	10.0
White cell count (cells per mmᶟ)	6000 -17500	30400	28570	16500
Differential count				
Neutrophils (%)	17 – 49	80.4	84	68.2
Lymphocytes (%)	66 – 77	17.2	15	29.1
Monocytes (%)	4 – 11	1.3	1.0	1.5
Eosinophils (%)	0 – 8	0.3	0.1	1.1
Basophils (%)	0 – 3	0.1	0.0	0.1
Platelet count (per mmᶟ)	150000 – 450000	478000	174000	275000
Sodium (mmol/liter)	135 – 145	141		132
Potassium (mmol/liter)	3.5 – 5.5	3.7		3.8
Creatinine (mmol/liter)	50 – 70	36		50
C – reactive protein (mg/liter)	< 8.0	27.3		
Aspartate aminotransferase (IU/liter)	19 – 25	22		24
Alanine aminotransferase (IU/L)	19 – 25	12		9

## Discussion

This three-year-old child initially presented with sub-acute respiratory symptoms, fever, weight loss and right upper lobe opacification. Pulmonary tuberculosis was considered because it is endemic in our country [1,2]. TB diagnosis is difficult in children and frequently relies on an algorithm relying on characteristic history, typical clinical signs and suggestive radiograph findings [1]. When the child failed to respond to anti-tuberculous therapy and developed the chest wall mass with draining sinuses, invasive fungal infection was considered including invasive aspergillosis and actinomyces. The incidence of invasive aspergillosis in developing countries is not known due to limited diagnostics and paucity of data [3]. Data for the paediatric population are even more scarce, as most of the reported cases are in adults. The commonest risk factor is a cavitary lung lesion, which in developing countries is often due to tuberculosis. There are reports of aspergilloma occurring in the context of chronic cavitary pulmonary aspergillosis [4]. Most cases of invasive aspergillosis in developing countries are due to *Aspergillus fumigatus*, which is ubiquitous and thrives in a hot, humid environment. The environmental load of conidia is increased by construction or renovation. The respiratory tract is the usual port of entry and the most common site of infection. The host's immune status influences the extent of mycelia colonization and tissue invasion. Risk factors include neutrophil defects, prolonged neutropenia, high dose corticosteroids, immunosuppressants, bone marrow and solid organ transplants. Newly recognized risk factors are reactive airway disease and allergic bronchopulmonary aspergillosis [5]. Antibiotics are a recognized risk factor for fungal infection, and third generation cephalosporins, carbapenems and glycopeptides in particular have been associated with increased risk of developing invasive *aspergillus* sp. infection [5]. This child did not demonstrate a clinical pattern consistent with congenital or acquired immune deficiency, nevertheless a deeper evaluation including neutrophil function would have been warranted. The presenting symptoms and radiological features may be indistinguishable from tuberculosis [6], and diagnosis may rely on histology as in our case. The drug of choice for treatment of invasive aspergillosis is voriconazole [7], which has been proven to be safe and effective in children. It is an expensive drug that is not readily available, and the patient was started on amphotericin B, as his renal function was normal. Early surgical intervention is recommended for some invasive pulmonary aspergillosis patients as it reduces the fungal burden and shortens the duration of antifungal therapy [4,7].

## Conclusion

This case highlights the importance of considering less common diagnoses in our setting particularly when there is poor response to empiric anti-tuberculous therapy. Among these considerations are deep fungal infections and therefore timely and aggressive diagnostic evaluation should be undertaken.
